# Fulminant Hepatic Failure in Dengue Fever Without Plasma Leakage: A Case Report

**DOI:** 10.7759/cureus.23964

**Published:** 2022-04-08

**Authors:** Vaishnavi Arunpriyandan, K T Sundaresan

**Affiliations:** 1 General Medicine, University Medical Unit, Teaching Hospital Batticaloa, Batticaloa, LKA; 2 Clinical Medicine, University Medical Unit, Teaching Hospital Batticaloa, Batticaloa, LKA

**Keywords:** acute liver failure (alf), therapeutic plasma exchange (tpe), lactic acidosis, hyperferritinaemia, hepatic encephalopathy, plasma leakage, fresh frozen plasma, n-acetyl cysteine, dengue fever

## Abstract

Dengue is an important arboviral disease in the tropics and subtropics. Although mild to moderate elevation of liver transaminases is a common phenomenon, dengue infection leading to hepatic failure is a rare complication in adults. We present a case of dengue fever in a young adult, leading to the rare complication of acute liver failure, without dengue shock syndrome. We tried evidence-based management with therapeutic plasma exchange, which led to a significant improvement in liver function.

## Introduction

Dengue fever is the most common mosquito-borne arboviral infection globally and is transmitted by *Aedes aegypti* and *Aedes albopictus* [1]. It is an endemic disease in Southeast Asia, including Sri Lanka. Four serotypes of the virus causing dengue fever have been identified, namely DEN 1 to DEN 4. Dengue fever has an extended clinical spectrum from asymptomatic infection to dengue fever, dengue hemorrhagic fever, and the most lethal dengue shock syndrome [1,2]. A severe form of the disease can involve several organs including the liver, brain, and kidney, and lead to fatal outcomes. Pathogenesis underlying severe illness is complex, but there is some evidence for cytokine storms and high levels of viremia to be associated with severe organ involvement. The liver is the commonly affected organ showing a spectrum of involvement extending from an asymptomatic liver enzyme elevation to the occurrence of acute liver failure (ALF) [2]. The pathophysiology of liver damage is yet to be completely understood. However, there are several mechanisms demonstrated including direct viral damage, immunological injury, and hypoxic injury due to reduced hepatic perfusion during shock [2,3]. Evidence-based guidelines are relatively less in managing ALF in dengue. In addition to N-acetyl cysteine (NAC), treatment modalities gaining importance in the recent past are steroids and therapeutic plasma exchange (TPE) [4].

## Case presentation

A 16-year-old male presented with a high fever for three days to a hospital in the eastern territory of Sri Lanka. He also complained of myalgia, arthralgia, and retro-orbital pain. There was no history of mucocutaneous bleeding or features of upper and lower respiratory infection. There was no significant past medical history including the use of hepatotoxic drugs, alcohol abuse, or past dengue infection.

On arrival, he was afebrile, conscious, and rational and was not pale or icteric. He was also hemodynamically stable with a blood pressure of 130/80 mmHg and a pulse rate of 88 bpm. There was no postural drop or tachycardia. He had no evidence of plasma leakage and the other system examinations were normal including the optic fundus. There were no rashes or lymphadenopathy.

Full blood count on admission showed white blood cells (WBCs) of 3 × 10^9^cells/L with lymphocytes of 1.37 × 10^9^/L and neutrophil of 2.20 × 10^9^/L and hemoglobin of 14.3 g/dL, hematocrit 43.1%, and platelet of 90 × 10^9^/L. Blood workup on renal function, liver function, and urine full report was within normal range initially. He was managed as possible dengue fever, which was confirmed by serum dengue non-structural protein 1 (NS1) antigen on day 4 of illness, which is the same day he got admitted. Reverse transcriptase-polymerase chain reaction (RT-PCR) and antibodies for COVID-19 infection were negative.

He was relatively well in the ward during the initial 36 hours, with routine supportive care and the standard fluid therapy according to the national dengue management guidelines. During these 36 hours, the clinical parameters remained within the normal limits.

At 36 hours of admission, he suddenly became restless. There were no bleeding manifestations or postural drop in blood pressure. He was otherwise stable with normal capillary glucose and hemodynamic parameters. Full blood count showed WBC 4.17 × 10^9 ^cells/L (neutrophil 65%, lymphocytes 32%), hemoglobin 14.6 g/dL, hematocrit 44.8%, and platelet count of 27 × 10^9^ cells/L. Liver biochemistry was as follows: alanine aminotransferase 546  U/L, aspartate aminotransferase 1080 U/L, alkaline phosphatase 144 U/L (range: 46-116), albumin 37 g/L, globulin 40 g/L, and total bilirubin 11.4 μmol/L. His prothrombin time/internationalized ratio was raised to 3.45. He was also found to have severe lactic acidosis with a lactate level of 4 mmol/L and a pH of 7.3. His other biochemical investigations such as renal function tests, serum calcium, and venous blood glucose were within the normal range (Table 1). Abdominal ultrasound showed no evidence of plasma leakage.

**Table 1:** List of investigations done according to Dengue monitoring protocol TPE, therapeutic plasma exchange; FBC, full blood count; HCT, hematocrit; WBC, white blood cell count; Plt, platelets; AST, aspartate aminotransferase; ALT, alanine transaminase; PT/INR, prothrombin time/internationalized ratio.

	Day 1	Day 3		12:00 am	Day 4		Day 5		Day 6		Day 7		Day 8		Day 9	Normal values
	8 pm	8 am	8 pm		8 am	8 pm	8 am	8 pm	8 am	8 pm	8 am	8 pm	8 am	8 pm	8 pm	
					1st TPE		2nd TPE		3rd TPE							
WBC	3 × 10^9^	3.4 × 10^9^	4 × 10^9^	5 × 10^9^	14 × 10^9^	15 × 10^9^	17.4 × 10^9^	16.7 × 10^9^	21 × 10^9^	22 × 10^9^	13 × 10^9^	16 × 10^9^	12 × 10^9^	7.77 × 10^9^	13120 × 10^9^	4.5-11.0 × 10^9^/L
Hb	14 g/dL	15 g/dL	15 g/dL		19 g/dL	19 g/dL	19 g/dL	10 g/dL	12 g/dL	11 g/dL	9 g/dL	10 g/dL	8 g/dL	8 g/dL	8 g/dL	13.5-17.5 g/dL
HCT	43%	44%	45%		57%	56%	55%	30%	34%	34%	25%	30%	26%	25%	26%	40-54%
Plt		86 × 10^9^/L	67 × 10^9^/L		18 × 10^9^/L	33 × 10^9^/L	23 × 10^9^/L	67 × 10^9^/L	57 × 10^9^/L	55 × 10^9^/L	28 × 10^9^/L	41 × 10^9^/L	25 × 10^9^/L	12 × 10^9^/L	88 × 10^9^/L	150-400 × 10^9^/L
AST	21 U/L	42 U/L		69 U/L	604 U/L	1080 U/L	805 U/L	247 U/L	112 U/L	60 U/L	92 U/L	63 U/L	50 U/L		65 U/L	7-56 IU/L
ALT	20 IU/L	26 IU/L		43 IU/L	546 IU/L	670 IU/L	643 IU/L	249 IU/L	120 IU/L	60 IU/L	71 IU/L	42 IU/L	44 IU/L		86 IU/L	7-45 IU/L
PT/INR		1.6		3.35	2.47	2.34	1.99	1.65	1.77	1.88	1.66	1.88	1.18	1.2	1.11	0.8-1.1
pH				7.19	7.39	7.4	7.37	7.39	7.43	7.4	7.4	7.19	7.35	7.4		7.35-7.45
pCO_2_				58 mmHg	35 mmHg	33 mmHg	41 mmHg	44 mmHg	38 mmHg	43 mmHg	43 mmHg	50 mmHg	56 mmHg	60 mmHg		35-45 mmHg
pO_2_				47 mmHg	105 mmHg	203	100 mmHg	51 mmHg	178 mmHg	191 mmHg	191 mmHg	63 mmHg	65 mmHg	65 mmHg		60-100 mmHg
Lac				3.9	3.1	2.6	2.6	2.2	1.9	1.4	1.4	1.8	1.9	1.7		<2
Serum ferritin	100 ng/mL			19,900 ng/mL		10,200 ng/mL		5400 ng/mL		850 ng/mL		470 ng/mL	200 ng/mL			20-250 ng/mL

He was immediately started on an ALF regime and methylprednisolone as well as a blood transfusion suspecting concealed bleeding. Despite the treatment, his consciousness (Glasgow Coma Scale) continued to deteriorate with persistent acidosis. His serum ferritin was 19,900.

He was immediately transferred to the intensive care unit. At this point of care, both clinical and biochemical parameters lead to a diagnosis of ALF with grade III hepatic encephalopathy, evidenced by altered liver biochemistry with coagulopathy, severe lactic acidosis, and altered level of the sensorium. He was started on intravenous NAC infusion and intravenous methylprednisolone 1 g daily for three days. None of his radiological, laboratory, or clinical parameters showed any evidence of plasma leakage throughout the illness.

**Figure 1:**
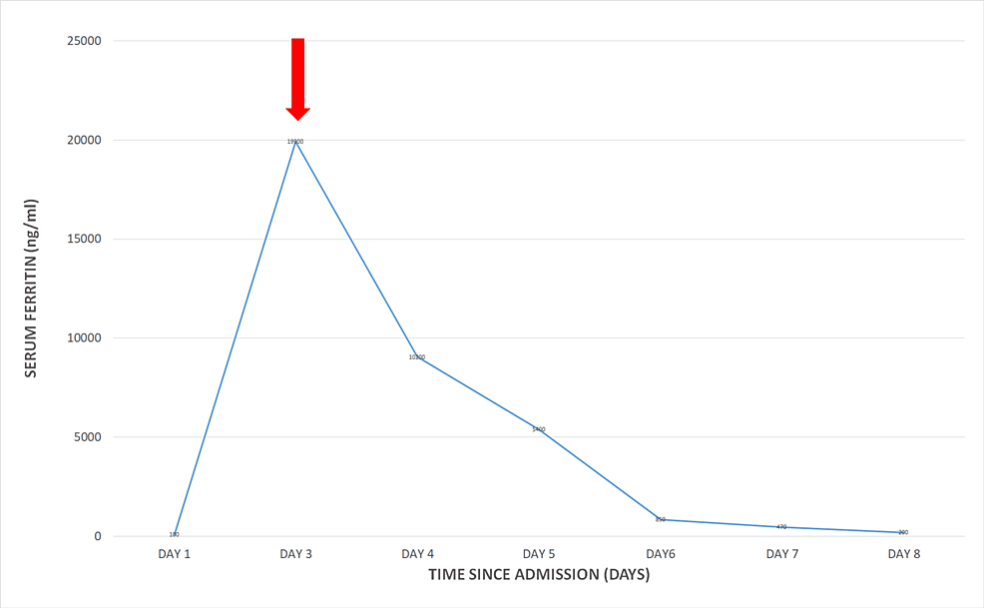
Drop of serum ferritin level with therapeutic plasma exchange Red arrow shows the time of patient deterioration as well as the initiation of therapeutic plasma exchange.

**Figure 2:**
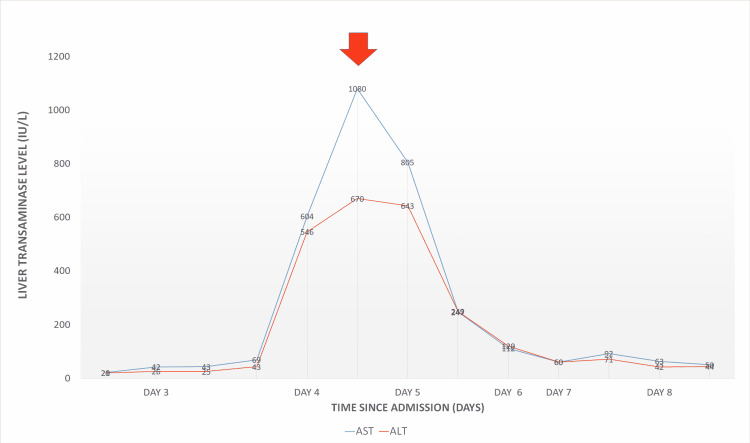
Change in liver transaminases with therapeutic plasma exchange ALT, alanine aminotransferase; AST, aspartate aminotransferase. Red arrow shows the initiation of therapeutic plasma exchange.

Except for the altered level of consciousness, the patient maintained stable vitals without going into the stage of dengue shock syndrome. He has no history of intake of hepatotoxic medicines such as non-steroidal anti-inflammatory drugs, paracetamol in supra-therapeutic doses, or herbal medicine known to cause liver damage. He was intubated due to a reduced level of consciousness. The next day his liver functions started to deteriorate with worsening acidosis with a remarkable rise in serum ferritin. A multidisciplinary team meeting was carried out and then he was started on three cycles of TPE, which significantly improved his liver function and acidosis in addition to the serum ferritin, which is an important marker of liver injury in dengue. Figures 1, 2 show the significant improvement of serum ferritin and liver enzymes with the initiation of TPE, respectively.

Coagulopathy was appropriately managed with fresh frozen plasma and cryoprecipitate. His biochemical and hemodynamic parameters improved. We were able to wean the patient off from supportive care eventually. While our team was planning to wean off supportive care completely, the patient suddenly developed bleeding from multiple sites such as intraventricular hemorrhage, gastrointestinal bleeding, and, finally, pulmonary hemorrhage, which was beyond the point of resuscitation.

## Discussion

Severe dengue (grade IV) is a disease of high mortality. Liver involvement is a very crucial feature seen in dengue infection [2]. Dengue virus has been shown to induce apoptosis of human hepatocyte cells. The exact mechanism by which this hepatic injury occurs is still unclear. Yet, T cell-mediated host immunity and the underlying cytokine storm is the postulated mechanism, which is often labeled as cytokine “Tsunami” [2,3].

Treatment principles such as NAC and prevention of hypoperfusion or dengue shock syndrome may play a certain role in mitigating the process, but none are proven to be beneficial in all cases. There are only a few therapeutic options available in the current era for severe liver disease [5]. ALF often results in multi-organ dysfunction including cardiovascular instability, renal failure, brain edema, and death due to irreversible shock. Coagulopathy and subsequent bleeding manifestations also play a key role in pathogenesis as in our patients [2,3].

Liver transplantation is the only proven method to rescue patients with ALF. Concerning the limited number of donors, it is impractical to provide liver transplantation to all patients with ALF. Therefore, new methods should be developed to halt the progression of hepatocyte injury. The TPE has been shown to modulate the innate immunity through which it improves liver transplant-free survival and ultimately the overall outcome. This hypothesis has been well studied in a recent randomized controlled trial by Larsen et al. [5,6]. Patients of dengue with hyperferritinemia-related multiorgan failure have been tried on TPE with variable success. WHO SEARO (World Health Organization in South-East Asia) guidelines also recommend TPE as a therapeutic option in severe dengue patients with hepatic encephalopathy, who deteriorate despite standard medical treatment [6]. It will be more beneficial if there are methods to identify patients who are at risk of developing ALF.

Guidelines on evidence-based management are sparse in the management of dengue leading to ALF. There are case series that showed substantial improvement in liver function following TPE in ALF. Two out of three patients survived following TPE in liver failure caused by dengue infection in Thailand as well as in one case from Sri Lanka [4,7]. Therefore, we used this evidence-based management in this patient, which showed significant improvement in liver functions. At the same time, we noticed that an early decision on TPE is crucial, as once the patient developed complications of ALF, such as coagulopathy, brain edema, and acute renal failure, mortality is inevitable [4]. In our patient, though the liver function and lactate improved with TPE, there was a multiorgan failure at the time we initiated TPE, which was beyond the point of resuscitation. But in cases where only liver failure is present, early initiation of TPE has improved mortality and morbidity. In our experience, serum lactate, lactate dehydrogenase, and especially higher levels of serum ferritin are the best markers of impending liver damage [4,8]. Vigilant monitoring is the key to identifying high-risk patients. Early initiation of treatment can save young lives.

## Conclusions

Post-dengue ALF has been fatal in the majority. But there are incidences where TPE has been a lifesaving treatment in severe dengue associated with hyperferritinemia. Further studies on the eligibility and timing of TPE in dengue will be beneficial in the future.
